# Proteomic profiling of high risk medulloblastoma reveals functional biology

**DOI:** 10.18632/oncotarget.3927

**Published:** 2015-04-23

**Authors:** Jerome A. Staal, Ling San Lau, Huizhen Zhang, Wendy J. Ingram, Andrew R. Hallahan, Paul A. Northcott, Stefan M. Pfister, Robert J. Wechsler-Reya, Jessica M. Rusert, Michael D. Taylor, Yoon-Jae Cho, Roger J. Packer, Kristy J. Brown, Brian R. Rood

**Affiliations:** ^1^ Center for Cancer and Immunology Research, Children's National Medical Center, Washington DC, USA; ^2^ UQ Child Health Research Centre, The University of Queensland and Queensland Children's Medical Research Institute, Children's Health, Queensland, Australia; ^3^ Division of Pediatric Neurooncology, German Cancer Research Center, Heidleberg, Germany; ^4^ Sanford-Burnham Medical Research Institute, La Jolla California, USA; ^5^ Department of Neurosurgery, Hospital for Sick Children, Toronto, Canada; ^6^ Department of Neurology and Neurological Sciences, Stanford University School of Medicine, Stanford, USA; ^7^ Center for Neuroscience and Behavioral Medicine, Children's National Medical Center, Washington DC, USA; ^8^ Center for Genetic Medicine, Children's National Medical Center, Washington DC, USA

**Keywords:** medulloblastoma, proteomics, cancer, cMYC, glycolysis

## Abstract

Genomic characterization of medulloblastoma has improved molecular risk classification but struggles to define functional biological processes, particularly for the most aggressive subgroups. We present here a novel proteomic approach to this problem using a reference library of stable isotope labeled medulloblastoma-specific proteins as a spike-in standard for accurate quantification of the tumor proteome. Utilizing high-resolution mass spectrometry, we quantified the tumor proteome of group 3 medulloblastoma cells and demonstrate that high-risk MYC amplified tumors can be segregated based on protein expression patterns. We cross-validated the differentially expressed protein candidates using an independent transcriptomic data set and further confirmed them in a separate cohort of medulloblastoma tissue samples to identify the most robust proteogenomic differences. Interestingly, highly expressed proteins associated with MYC-amplified tumors were significantly related to glycolytic metabolic pathways via alternative splicing of pyruvate kinase (PKM) by heterogeneous ribonucleoproteins (HNRNPs). Furthermore, when maintained under hypoxic conditions, these MYC-amplified tumors demonstrated increased viability compared to non-amplified tumors within the same subgroup. Taken together, these findings highlight the power of proteomics as an integrative platform to help prioritize genetic and molecular drivers of cancer biology and behavior.

## INTRODUCTION

Remarkable progress has been made in defining the genomic aberrations that underlie pediatric brain tumors. In regard to medulloblastoma (MB), the most common malignant pediatric brain tumor, what was originally perceived to be a single entity is now known to consist of at least four distinct subgroups with significantly different clinical outcomes and therefore warranting individual therapeutic consideration [1]. Group 3 MB has the worst outcome with independent studies reporting between 40 to 50% overall survival [1-3]. Metastases and myc amplification predict poor prognosis in Group 3 MB despite aggressive therapy [4]. As a result, there is an imperative to improve therapy for patients with group 3 tumors, with a particular focus on high risk cases [5].

Although transcript-based profiling technologies enable separation of subgroups based on gene expression, it is often difficult to interpret such signatures with respect to the biology of the disease. This is largely because these gene signatures do not incorporate important functions downstream of gene transcription, including splice isoform switching, selective translation and post-translational modification [6]. Proteomics provides a unique complementary platform to precisely characterize altered signal transduction in cancer cells, examine cellular processes and identify potential therapeutic targets. Recent advances in mass spectrometry (MS) technology have improved protein identification and quantitation due to increased sensitivity, accuracy and speed of analysis [7-9]. The stable-isotope labeling of amino acids in cell culture (SILAC) method is a particularly accurate quantitative technique that has recently been used to identify diffuse large B-cell lymphoma subtypes and quantify diverse human tumor proteomes [6, 10].

SILAC involves incorporating amino acids constructed using the isotopes ^13^C and/or ^15^N into proteins. This increase in mass allows otherwise chemically indistinguishable peptides from different samples to be individually quantified by the mass spectrometer, even when pooled. Specifically, a protein lysate from an experimental sample can be pooled with a known quantity of an isotope-labeled protein lysate, fractionated and digested into peptides. The mass spectrometer can then quantitate both the naturally occurring ^12^C- and ^14^N-containing as well as the heavy analytes as a ratio. Performing this process iteratively with multiple experimental samples is a proven method for relative quantification of peptides, and thus proteins, between those samples [12].

We present here the first in depth proteomic profiling of MB using multiple SILAC labeled cell lines as an internal standard for agnostic tumor protein quantification. We specifically investigate proteins with differential abundance between low (non-MYC amplified) and high risk (MYC amplified) group 3 MB tumors [11], and identify targets that provide insight into the tumor biology of this aggressive subgroup. In summary, our data demonstrates the potential of SILAC-based proteomics to reliably quantify global proteome differences and to complement the current understanding of genomic abnormalities in medulloblastoma.

## RESULTS

### Super-SILAC proteomic analysis enables accurate quantification of subgroup specific proteins

**Figure 1:**
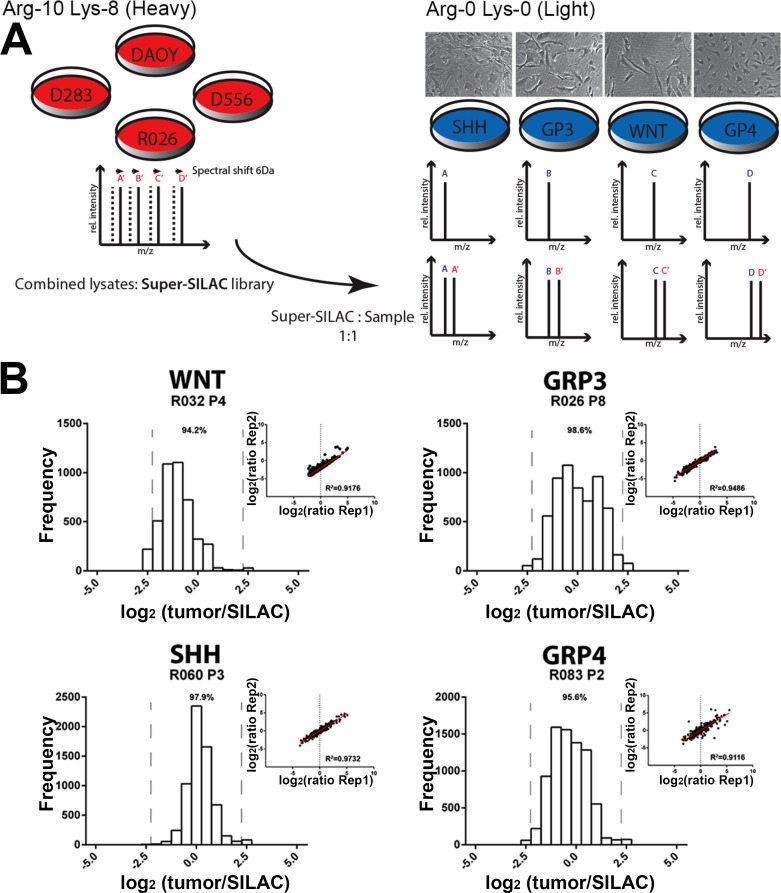
Effective quantification of human primary tumor cells using a super-SILAC reference standard **A.** Experimental scheme of quantification analysis using mixed lysates of multiple MB tumor cell lines. Lysates of labeled cells (Lys-8, ^13^C_6_^15^N_2_-Lysine; Arg-10, ^13^C_6_^15^N_4_-Arginine) are mixed with tumor lysate (at a 1:1 ratio) and analyzed by high resolution liquid chromatography-MS/MS. **B**; Histograms of the ratios between the tumor protein and our super-SILAC reference, and a comparison of ratios (*r* = Pearson correlation coefficient) between replicates demonstrates the high quantification accuracy of our technique. Superior accuracy is achieved when quantified proteins (proportion indicated by percentage above histogram) lie within four-fold ratio between tumor and super-SILAC reference [10]

Current antibody based proteomic techniques are useful tools, but only once a small set of target proteins have been identified. Mass spectrometry, by contrast, provides broad proteome coverage, but has historically been plagued by low sensitivity, inaccurate quantitation and technical variability. For this study, we utilized a novel SILAC based mass spectrometry technique to reproducibly quantify proteome differences in human primary tumor cells. The SILAC approach involves the use of a set of stable isotope-labeled labeled peptides that can be spiked, at known amounts, into test samples and used as an internal reference standard for accurate quantification of proteins by mass spectrometry [12]. We developed a unique SILAC reference standard comprised of three labeled MB tumor cell lines (DAOY, D556, D283) and a labeled low passage primary MB tumor cell culture (R026; group 3 non-metastatic tumor) (Figure 1A). This mixture is termed super-SILAC as it is a superset of SILAC cell lines and has previously been shown to achieve superior quantification accuracy compared to single SILAC-labeled cell line standards [10]. Creating the reference from a pool of labeled protein lysates from multiple MB primary and established cell lines broadens proteome coverage to increase the number of quantifiable proteins in the experimental MB samples.

To test the proteomic coverage and accuracy of our unique super-SILAC standard when studying heterogeneous subsets of MB, we evaluated primary lines representing the 4 molecular subgroups of MB. We identified between 1400-1900 proteins per primary MB cell line; ≥94.5% of all peptides fell within a 2.5-fold ratio of tumor to SILAC standard (Figure 1B). This narrow ratio distribution contributed to significantly higher quantification accuracy with all MB cells displaying a ≥0.92 Pearson correlation coefficient of variation between triplicate analyses (Figure 1B). Approximately half of all proteins identified (45-56%), using stringent peptide probability scores and false discovery rate filters (see *Methods and materials*), were shared by all MB subgroups, with Group 3 and Group 4 displaying the greatest similarity in their proteomes (Figure S1). There was no significant difference in quantification accuracy and proteome coverage across the MB subgroups (> 80% of proteins overlapped between the standard and each sample; Figure S1), demonstrating the ability of our super-SILAC standard to function across diverse subgroups of MB. In summary, our unique MB super-SILAC standard, together with high-resolution mass spectrometry, was able to accurately and reproducibly quantify a large set of proteins across all MB tumor subgroups. Having illustrated the effectiveness of our super-SILAC standard, we next evaluated the proteomic differences between MYC amplified versus non-amplified tumors.

### Differential proteome patterns in MYC amplified versus non-MYC amplified Group 3 tumor cells

**Figure 2:**
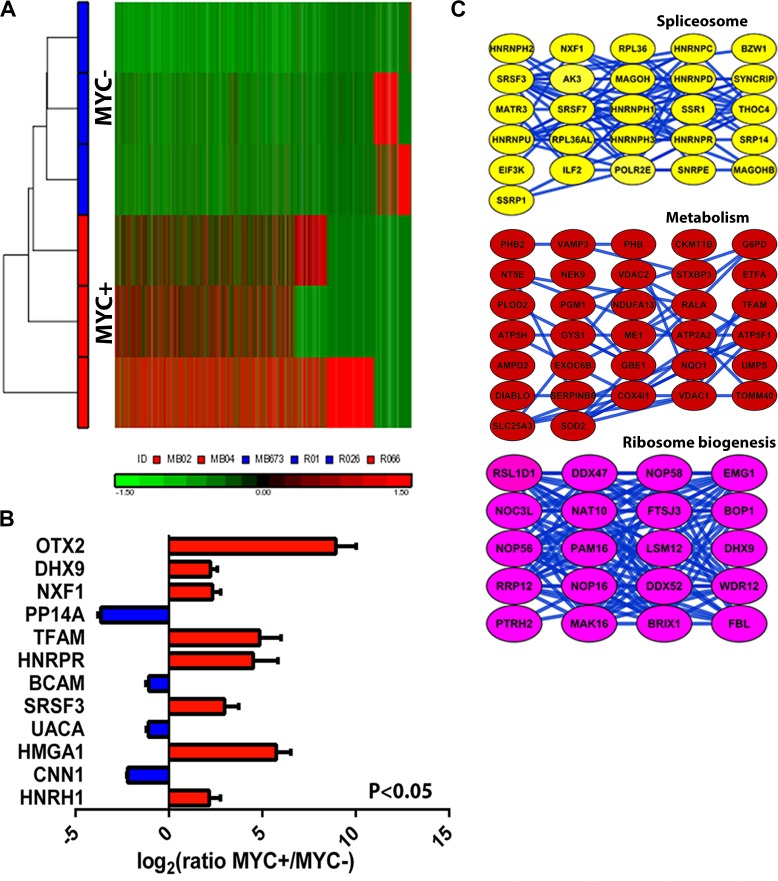
Differential proteome expression patterns between MYC-amplified versus non-amplified tumors **A.** Unsupervised hierarchical clustering (normalized log_2_ and standardized to mean signal = 0 and standard deviation = 1) of protein expression profiles from six Group 3 primary MB cells **B.** Differential protein expression drives segregation of tumor cells with MYC-amplification, which is not seen when similar analysis is conducted using transcriptome data (see FigS3). **B, C.** Top up-regulated proteins and predicted functional pathways in MYC-amplified tumors. STRING-generated protein-protein interaction pathways revealed significant (*P*, 0.05; *t*-test) connectivity in alternative splicing, ribosome biogenesis and metabolism pathways. Error bars represent standard deviation between the cultures in each subgroup (*n* = 6).

Group 3 MB has the worst overall survival (< 50%) and transcriptional profiling analyses have not revealed actionable targets. MYC amplification in this subtype is significantly associated with metastasis and poor survival, yet little is known regarding the cellular and molecular mechanisms that produce this aggressive phenotype. Using our super-SILAC standard we explored the global proteome differences between human MYC amplified (MYC+, *n* = 3) versus non-MYC amplified (MYC−, *n* = 3) Group 3 primary tumor cells. Interestingly, unsupervised hierarchical clustering of the median protein expression values of all quantified proteins resulted in segregation by MYC amplification status (Figure 2A-2B). Focusing on only significant protein changes (*p*-value with FDR correction < 0.001), we identified 185 proteins (Table S1) that were differentially expressed between MYC amplified and non-amplified tumors (Figure 2C; Table S1). Intriguingly, some of the highest differentially expressed proteins, such as orthodenticle homeobox 2 (OTX2) and DEAH box helicase 9 (DHX9), have previously been shown in genomic studies to be strongly associated with Grp3/4 MB [1, 2, 12]. To determine the functional significance of these proteins, we utilized the Search Tool for the Retrieval of INteracting Genes/Proteins (STRING) database of physical and functional interactions [13]. In the global STRING-generated protein-protein network, several complexes and cellular functions formed prominent tightly connected clusters (Figure S2). Cellular processes such as RNA splicing and cellular metabolism were particularly enriched in MYC+ tumors compared to MYC−. These MYC+ associated cellular pathways prominently featured heterogeneous ribonucleoproteins (HNRNPAB, HNRNPH1, and HNRNPC) and serine/arginine-rich splicing factors (SRSF2, SRSF7), indicative of post-transcriptional modification pathways. We further validated our proteomic data by western blot analysis, confirming the significantly differential expression of spliceosome associated proteins (Figure S3). Western blot analysis of Grp3 tumor cell lines was consistent with our super-SILAC mass spectrometry observations.

To determine the relationship between our proteomic findings and the transcriptomic landscape, we performed an orthogonal analysis of independent gene expression data from the largest single human MB study (GEO accession number GSE37385) [14] to determine the differential gene expression between ten MYC+ and ten MYC− human Grp3 MB tumors. There was limited concordance between protein and mRNA variation (8% similarity with differentially expressed gene transcripts with *p* < 0.05). Interestingly, interaction pathways generated from these common proteogenomic targets revealed strikingly similar pathways to those seen in our proteomic data alone (Figure S3;. These results highlight the strength of proteomics to help validate and prioritize the broad output of genomic studies to determine those most closely associated with cancer function/biology.

### Cross-validation of proteomic MYC-signature in independent human tumor tissue samples

**Figure 3:**
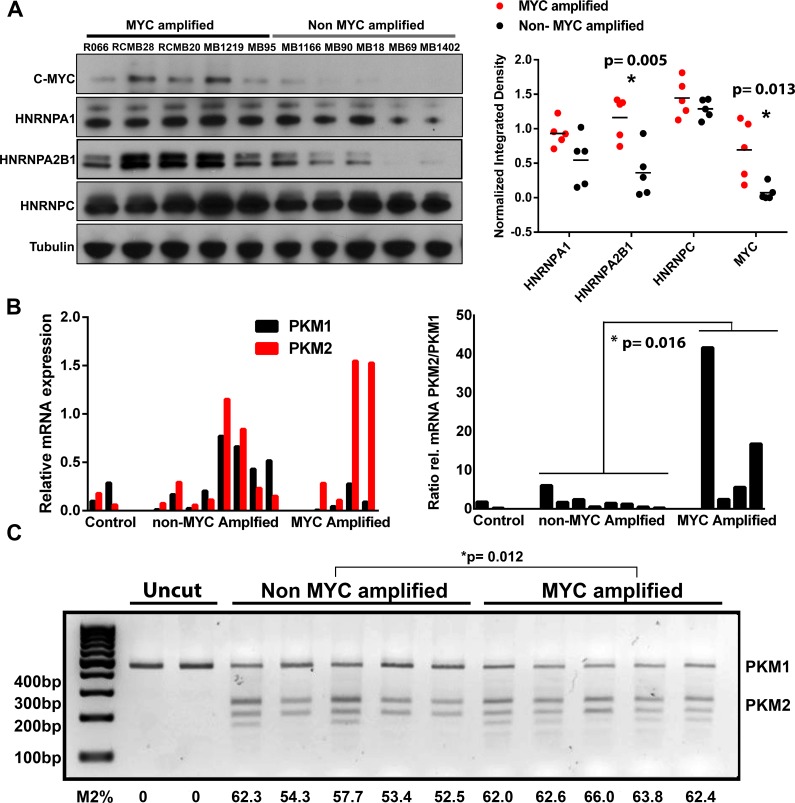
Validation of differential protein expression in independent human MB tissue samples and evidence for increased alternative splicing of PKM **A.** Confirmation of proteomic alterations in independent human MB tissue samples using western blot. Significant (*t*-test) alterations in splicing associated factors were conserved between low passage primary tumor cell cultures and human tissue samples. **B.** Quantitative real-time PCR in human MB tissue and cell cultures reveals increased alternative splicing of PKM to produce the pro-glycolytic PKM2 isoform in MYC amplified tumors. **C.** cDNA samples from independent human tissue samples were subjected to PCR amplification using primers amplifying a 442 bp exon 8-11 region common to PKM1 and PKM2. Following incubation with PstI, the uncleaved (PKM1, 442 bp) and cleaved (PKM2, 246 and 196 bp) amplification products were separated by electrophoresis and quantified, with total signal set at 100 for each lane (Lane 1 uncut MYC-, Lane 2 uncut MYC+). There is significantly higher splicing of mRNA which is indicative of higher PKM2 isoforms.

Proteomic differences in our primary tumor cultures could result from adaptations to an *in vitro* culturing system and may not faithfully reflect protein expression in human tumor tissues. To validate proteomic differences shown in our super-SILAC system, we used western blotting on an independent group of human tumor tissue samples (*n* = 10; see Table S2). Greater proteomic variability was observed between human tumor tissue samples than in culture, however significant differences in HNRNPA2/B1 (*p* = 0.005) and MYC (*p* = 0.013) were still detected between MYC+ and MYC− tissues (Figure 3A). There were no significant differences in HNRNPA1 (*p* = 0.068) and HNRNPC (*p* = 0.281), which were significantly higher in MYC+ primary cultures compared to MYC-. These results support our *in vitro* finding of altered expression of splicing factors in MYC+ group 3 human MB tissues.

We next determined whether this significant increase in splicing factors was associated with increased splicing of their targets. Notably, HNRPNPA1, HNRPNPA2 and PTB have previously been shown to regulate alternative splicing of pyruvate kinase pre-mRNA (PKM) resulting in increased levels of the embryonic pyruvate kinase isoform, PKM2 [15]. Importantly, PKM2 promotes aerobic glycolysis, whereas the adult isoform, PKM1, promotes oxidative phosphorylation. These two isoforms result from mutually exclusive alternative splicing of PKM pre-mRNA leading to the inclusion of either exon 9 (PKM1) or exon 10 (PKM2). We therefore used quantitative PCR analysis using primers specific for PKM exon 9 or PKM exon 10 to determine shifts in the PKM2/PKM1 ratio. Real time PCR (RT-PCR) revealed increased levels of the PKM2 isoform in the MYC+ MB tumors compared to MYC− tumors, and a subsequently higher PKM2/PKM1 ratio (Figure 3B). Although there was high variability in the PKM2/PKM1 ratios among the MYC+ tumor tissues, the mean ratio was significantly higher (*P* = 0.016, Mann-Whitney test) than in the non-amplified MYC tumors. This was further confirmed in a partially overlapping human MB tumor tissue set using a restriction enzyme assay to assess the proportion of PKM1 and PKM2 (Figure 3C; *P* = 0.012, *t* test).

### Alterations in glycolytic metabolism in MYC amplified MB tumor cells

**Figure 4:**
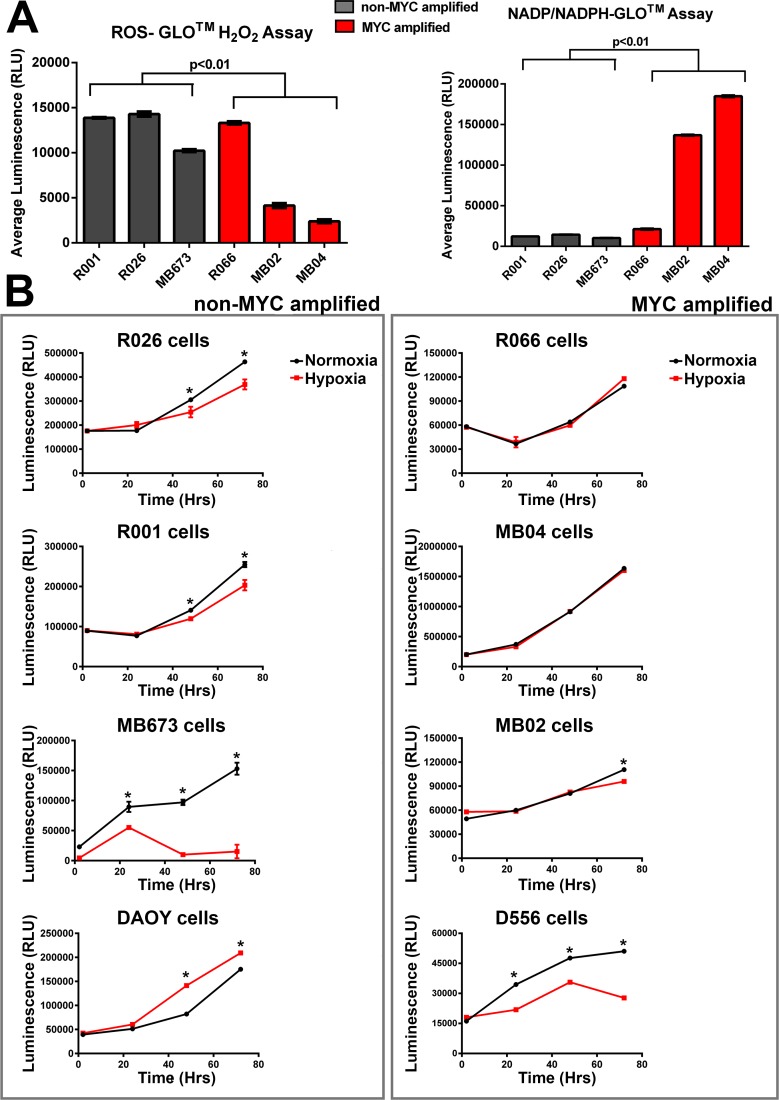
MYC-amplified tumor cells have significantly altered metabolic activity compared to the rest of Group 3 MB cells (non-MYC amplified) **A.** Although MYC amplified tumor cells have significantly lower reactive oxidative species (H_2_O_2_), they also have higher levels of total nicotinamide adenine dinucleotide phosphates (*t*-test; *p* < 0.01; error bars are standard deviation between subgroup cultures). **B.** ATP production under hypoxic conditions (48 hours, 0.1% O_2_) is significantly reduced in most (3/4) non-MYC amplified tumor cells, but is not perturbed in MYC-amplified tumors (3/4). Error bars are standard deviation of sample replicates; *t*-test,* denotes *P* < 0.05.

Alternative splicing of PKM plays an important role in determining the metabolic phenotype of mammalian cells. We set out to determine if the increased splicing factors and subsequent alternative splicing of PKM, resulting in a high PKM2/PKM1 ratio, correlated with glycolytic metabolism in these tumor cells. Intriguingly, MYC amplified tumor cells displayed a significantly higher production of total oxidized and reduced nicotinamide adenine dinucleotide phosphates (NADP+ and NADPH, respectively) when compared to non-amplified (MYC-) cells (*P* < 0.01; Figure 4A). High metabolic flux through glycolysis produces ATP and intermediates which subsequently generate NADPH and ribose for reductive biosynthetic reactions and nucleotide synthesis - both critical to a highly proliferative phenotype. Surprisingly, although increased reactive oxidative species (ROS) production is associated with up-regulation of glycolysis, we saw significantly lower levels of H_2_0_2_ in our MYC-amplified tumor cells (*P* < 0.01; Figure 4A). This ROS quantification was independent of cell survival and proliferation, as determined by cell viability assays after ROS measurements (Figure S4). As we also demonstrate significantly higher protein expression of superoxide dismutase (SODm) in MYC+ tumors (see Table S1), these results may suggest an adaptation to a phenotype resistant to acid-induced toxicity. Constitutive up-regulation of glycolysis is proposed to be an adaptation to hypoxia. To test this, we compared ATP production in cultures grown under hypoxic (< 1% O_2_) and normoxic conditions. All non-amplified MYC cultures, except for the DAOY cell line, produced significantly (*P* < 0.05) lower ATP when grown in hypoxic conditions (Figure 4B). Conversely, there were no significant differences in ATP production in MYC+ cells grown in a hypoxic environment, except for the D556 cell line, when compared to normoxia (Figure 4B). In summary, energy production was unperturbed in primary MYC+ MBs when cultured under hypoxic conditions.

## DISCUSSION

The results of this study demonstrate the power of quantitative proteomics as a platform to help discern molecular and genetic drivers of cancer. The application of proteomics is particularly useful when studying genetically ambiguous tumors like group 3 MB, which has the highest levels of recurrence and the worst overall survival rates [1, 3, 12]. Our proteomic findings, validated in clinical samples at the level of transcript and protein, provide new evidence of the biological effect of MYC amplification in group 3 MB. Specifically, we demonstrate alternative splicing of pyruvate kinase mRNA by HNRNPs and the subsequent altered metabolic phenotype in these tumors. Intriguingly, this may influence the bioenergetics of MYC amplified tumors and therefore confer a powerful growth advantage necessary for the evolution and metastasis of this aggressive cancer.

Our proteomics data highlight significant differences in the metabolism of MYC amplified versus non-amplified tumors. This pathway has not previously been studied in MB, possibly due to the hypothesis that MYC amplification was primarily associated with dysregulated cellular proliferation with a subsequent increase in metabolism. However, previous findings that MYC directly regulates genes involved in glucose metabolism and ribosome biogenesis (also supported in this study), suggests that the increased production of metabolic enzymes in growing cells is far from a passive phenomenon [16-19]. Enhanced metabolic capacity is essential for biomass accumulation and high-fidelity DNA replication in a growing cell. This enhanced capacity requires rewiring of the metabolic energetics of a resting cell into that of a growing and proliferating cell. To this effect, MYC stimulates virtually all of the genes involved in glycolysis [15, 20]. High metabolic flux through glycolysis provides growing cells with the building blocks for macromolecular synthesis and ATP production. Importantly, our results suggest that this shift in metabolism in MYC amplified tumors may likely influence the adaptation of these cells to a hypoxic microenvironment. This is supported by evidence that MYC induction of glutamine metabolism is important for cell survival under glucose and oxygen-deprived conditions [18, 20]. Taken together, we propose that this MYC-amplification associated glycolytic phenotype may confer a powerful growth advantage and influence the aggressive behavior of this tumor subtype.

It is important to note that myc amplification in this context defines a subgroup of tumors whose biology is not necessarily wholly defined by the transcriptional activity of MYC and its target genes. This is an important distinction that has been written about previously [21]. In fact, increased MYC expression (without MYC copy number variation) is also found in the WNT subgroup medulloblastoma tumors with the highest survival. The data presented here support the concept that there are significant differences in protein expression between MYC-amplified and non-amplified tumors, though whether this is directly related to increased MYC transcript expression is an open question. For example, two of the primary MYC amplified lines used to generate the proteomic data also harbor OTX2 amplifications. As OTX2 amplification is known to cooperate with MYC to amplify its transcriptional program, and OTX2 was the most differentially expressed protein found in the MYC+ group, it is possible that the proteomic phenotype here described is driven by the cooperation of these two oncogenes [22].

MYC amplification is more closely associated with group 3 MBs than any other group and makes an excellent prognostic marker in combination with established clinical risk factors [11]. However, transcriptional profiling has not revealed appreciable myc-dependent signaling pathways [2, 12] and next-generation sequencing in three independent studies yielded few recurrent mutations in this group [3, 23-25]. The proteomics perspective enhances and complements our current understanding of genomic drivers of cancer biology and provides a novel platform for exploring molecular pathways in medulloblastoma.

## MATERIALS AND METHODS

### Patients and samples

The use of human tissue (Table S2) was approved by the institutional review board of Children's National Medical Center (Washington DC, USA) (IRB 4932). Written informed consent was obtained at the time of surgical resection. De-identified MB tissue were obtained from Queensland Children's Tumor Bank (Brisbane, Australia), German Cancer Research Center (Heidleberg, Germany), Sanford-Burnham Medical Research Institute (La Jolla, USA), and Hospital for Sick Children (Toronto, Canada). Primary passage patient derived mouse xenograft cells were provided by Dr. Wechsler-Reya (Sanford-Burnham Medical Research Institute, La Jolla, USA).

### Cell lines and SILAC culture

Primary MB cell cultures R001, R026, R032, R060, and R066 (see Table S2 for details) were obtained from Queensland Children's Tumor Bank. The cell lines were cultured in ‘light’ DMEM: F12 media (Gibco) containing naturally occurring lysine and arginine and supplemented with 10% fetal bovine serum (Gibco), 1% glutamax (Gibco), 1% sodium pyruvate (Life Technologies, Grand Island, NY) and 1% penicillin/streptomycin/fungizone (supplier). MB002 and MB004 cell cultures were a kind gift from Dr. Jae Cho (Stanford University, Stanford, CA) and maintained in 1:1 DMEM/F12 (Gibco) and Neurobasal-A medium (Gibco) supplemented with B27 (Gibco), EGF (Fisher Scientific), fibroblast growth factor (Fisher Scientific), Heparin (Sigma-Aldrich), and leukemia inhibitory factor (Millipore). Established MB cell lines D556 and D283 were generously provided by Dr. Darrell Bigner (Duke University, Durham, NC) and the DAOY cell line obtained from the American Type Tissue Collection. These cell lines were SILAC-labeled by culturing in DMEM media (Life Technologies, Grand Island, NY) containing ‘heavy’ isotope-labeled essential amino acids, ^13^C_6_, ^15^N_2_-lysine (Lys-8) and ^13^C_6_, ^15^N_4_-arginine (Arg-10). The labeled amino acids were purchased from Cambridge isotope Laboratories (Andover, MA). Media was supplemented with 10% fetal bovine serum, 1% glutamax, 1% sodium pyruvate and 1% antibiotics as above. The primary cell line R026 was also SILAC labeled by culturing in DMEM:F12 media (Thermoscientific, Rockford, IL) where Lys-8 and Arg-10 replaced naturally occurring amino acids. Cell lines were cultured for at least 6 population doublings until fully labeled, as confirmed by mass spectrometry (> 99% incorporation, data not shown). All cell lines were maintained at 37°C and 5% CO_2_. It is important to note that of the cell lines used in our super-SILAC mix, D283 and D556 harbor cMYC gene copy number amplifications whereas DAOY and R026 do not.

Cell pellets from all unlabeled cell lines and SILAC-labeled cell lines and snap frozen tumor tissue were lysed in RIPA buffer (Millipore, Temecula, CA) containing 2% SDS (Lonza, Rockland, ME) and protease inhibitors (Thermoscientific, Rockford, IL). Lysates were incubated on a shaker at 4°C for 30 minutes and sonicated for 10 minutes followed by centrifugation. Snap frozen tumor tissue was homogenized with a handheld homogenizer then lysed using the same method. The supernatant was reserved and protein concentration was determined by BCA assay (Pierce Thermoscientific, Rockford, IL) at a wavelength of 562nm. The super-SILAC spike-in standard was created by mixing equal amounts of protein from each SILAC-labeled cell line (R026, DAOY, D556 and D283). Protein concentration was determined by BCA assay and aliquots of the standard were stored at −80°C until required.

### Super-SILAC standard and proteomics

Unlabeled primary cell lysates were mixed with the super-SILAC standard in a 1:1 ratio by protein content, as determined by BCA assay. All samples were desalted and buffer exchanged to Tris/HCl buffer using Micro bio-spin 6 columns (Bio-Rad, Hercules, CA). Protein concentration was determined by BCA assay and aliquots containing 80ug of total protein per sample run (up to 240ug for triplicate analysis) were dried by vacuum centrifugation. Protein pellets were resuspended in XT sample buffer containing Criterion XT reducing agent and denatured at 95°C for five minutes. Proteins were resolved by SDS-PAGE on Criterion XT 4-12% Bis-Tris gels (Bio-Rad, Hercules, CA) at 180 volts for 1 hour. The gels were fixed (50:5:45/methanol:aceticacid:water/v:v:v), stained with Bio-Safe Coomassie blue (Bio-Rad, Hercules, CA) and destained in H2O. 32 gel sections were excised from each sample run and the individual bands were processed for in-gel trypsin proteolysis as previously described^10^. Trypsin digestion was performed using 12.5ng/ul of mass spectrometry grade Trypsin (Promega, Madison, WI) diluted in 25mM NH_4_HCO_3_ solution. Peptides recovered from each band were dried by vacuum centrifugation and resuspended in 8ul of 0.1% TFA for mass spectrometry analysis.

### Mass spectrometry analysis

NanoLC MS and MS/MS were conducted using the Eksigent nanoLC 2D HPLC system (Eksigent Technologies, Inc., Dublin, CA) coupled to the LTQ-Orbitrap XL hybrid mass spectrometer (Thermo Fisher Scientific, San Jose, CA). Peptides from each band were injected via an autosampler (6uL) and loaded onto a Symmetry C18 trap column (5μm, 300 μm i.d. × 23 mm, Waters) for 10 min at a flow rate of 10 μL/min, water with 0.1% formic acid. The sample was subsequently separated by a C18 reverse-phase column (3 m, 200A, 100 μm × 15 cm, Magic C18, Michrom Bioresources) at a flow rate of 300 nL/min using an Eksigent nano-hplc system (Dublin, CA). The mobile phases consisted of water with 0.1% formic acid (A) and 90% acetonitrile (B). A 65 minute linear gradient from 5 to 60% B was employed. Eluted peptides were introduced into the mass spectrometer via Michrom Bioresources CaptiveSpray. The spray voltage was set at 2.2kV and the heated capillary at 200°C. The LTQ-Orbitrap-XL (ThermoFisherScientific) was operated in data-dependent mode with dynamic exclusion in which one cycle of experiments consisted of a full-MS in the Orbitrap (300-2000 m/z) survey scan in profile mode, resolution 30,000, and five subsequent MS/MS scans in the LTQ of the most intense peaks in centroid mode using collision-induced dissociation with the collision gas (helium) and normalized collision energy value set at 35%. Proteins were identified and quantified from spectral data using Integrated Proteomics Pipeline (IP2) version 1.01 software developed by Integrated Proteomics Applications, Inc. (http://www.integratedproteomics.com/). Files from each sample lane were searched against the forward and reverse Uniprot human database (UniProt release 2013_01 with 20,226 reviewed entries) for partially tryptic peptides allowing one missed cleavage, and possible modification of oxidized methionine (15.99492 Da) and heavy arginine (6.0201 Da) and heavy lysine (8.0142 Da). IP2 uses the Sequest 2010 (06_10_13_1836) search engine. Mass tolerances were set at +/− 50 ppm for MS and +/− 1.5 Da for MS/MS. Data were filtered based on a 1% protein false discovery rate and two unique peptides. All the bands from each lane were \summed in the analysis. Census software version 1.77, built into the IP2 platform, was used to determine the ratios of unlabeled and labeled peptide pairs using an extracted chromatogram approach. The distribution of ratios was plotted and correction factors applied to adjust for error in sample mixing. Data were checked for validity by using regression correlation better than 0.5 for each peptide pair. Peptide ratios were averaged to yield the protein ratio with standard deviation.

### Western blot

Protein lysates from MB primary cultures and frozen operative tissue were prepared in lysis buffer (50 mM HEPES, pH7.0, 150 mM NaCl, 2% SDS, 1 mM EDTA) supplemented with protease and phosphatase inhibitors (Roche). Proteins were separated on 4–20% Bis-Tris gradient polyacrylamide gels (Invitrogen) and transferred onto Immuno-Blot nitrocellulose membranes (Bio-Rad Laboratories). Membranes were then incubated in blocking buffer (1X TBS containing 5% milk and 0.05% Tween-20, 1 hour), probed overnight with antibodies specific for HNRNPA2/B1 (Cell Signaling, 1:1000), HNRNPA1 (4B10; Santa Cruz; 1:2000), HNRNPC1/C2 (4F4; Santa Cruz; 1:5000), cMYC (9E10; Santa Cruz; 1:500) and actin (Sigma; 1:2000).

### Quantitative RT-PCR

Total RNA was isolated from samples (Qiagen RNeasy Kit), treated with DNase I, reverse transcribed (Superscript II Reverse Transcriptase Kit, Invitrogen) and amplified (initial denaturing of 95°C for 3 min followed by 40 cycles at 95°C for 15 sec, 60°C for 15 sec, and 72°C for 30 sec) in triplicate using primers designed to bind to PKM1-specific exon 9 (F-ACCGCAAGCTGTTTGAAGAA and R-TCCATGAGGTCTGTGGAGTG) or PKM2-specific exon 10 (F-GAGGCCTCCTTCAAGTGCT and R-CCAGACTTGGTGAGGACGAT), sequences, respectively as previously described [26]. The mean Ct values for each set of amplifications were determined, after which the mean Ct value from triplicate qPCR reactions using primers for the housekeeping gene Actin were subtracted to derive a ΔCt value. Relative mRNA expression was expressed as 2^ΔCt^.

### PKM splicing assay

The PKM splicing assay used was conducted as previously described [15, 26]. Briefly, RNA isolated from normal or tumor samples (fixed or frozen) was reverse transcribed then amplified (98 °C for 3 min followed by 98°C for 20 sec, 65°C for 20 sec, 72°C for 30 sec for 34 cycles) using a forward primer that bound to shared PKM1/2 exon 8 and a reverse primer that bound to shared PKM1/2 exon 11 sequences [15]. The 442 bp PCR products representing both PKM1 and PKM2 transcripts were then digested with PstI and electrophoresed, after which the 442 bp (uncut PKM1-specific amplicon) and 246 bp and 196 bp (PstI-cleaved PKM2-specific amplicon) products were quantitated (Image-J software). Sham-digested PCR products were used as a control for the PCR amplification while PstI-digested amplification products derived using a PKM2 cDNA template were used as a control for restriction enzyme digestion.

### Network analysis for protein-protein interactions

The Uniprot identifiers of our highly increased proteins in MYC-amplified tumors (3-fold and higher; p-value with FDR correction < 0.01) were mapped to the Ensemble protein identifiers, and then searched against the STRING database version 9.1 [13] for protein-protein interactions. Only interactions between the proteins belonging to our MYC-amplification dataset were selected. STRING defines a metric called “confidence score” to define interaction confidence; we fetched all interactions for our acetylation dataset which had a confidence score ≥ 0.7 (high confidence).

### Genome-wide expression analysis

Previously published microarray expression and copy-number data^3^ were obtained from the Gene Expression Omnibus (GEO; GSE37385 and GSE37382). The expression data were obtained using the GEOImporter module in GenePattern. Z scores of gene expression values of genes within samples were calculated. The genes were mapped to ensemble identifiers and searched against the STRING database version 9.1 [13] to identify enriched interactions.

### Oxygen deprivation

Tumor cells were seeded in triplicate in 24 well plates (Corning Costar, Sigma Aldrich, St. Louis, MO) in appropriate medium (blank wells contained only media without cells). The normoxia set of plates were placed in an aerobic incubator (atmospheric) and the hypoxic/anoxic set were placed in a ProOx110/ProCo_2_/C-Chamber (Biospherix, Japan), which was equilibrated to 37°C in a humidified atmosphere of 5% CO_2_ and 0.1% O_2_. At the appropriate time, the cells were removed from the incubator and equal volumes of CellTiter-Glo® (Promega, Madison, WI) added to each culture and mixed for 2 min at room temperature as described per protocol. After allowing 10 min to stabilize the luminescent signal, 200μl of each of the lysates (including replicates) were placed in opaque-walled 96-well plates and the luminescence recorded (FLUOstar optima, BMG labtech, Ortenberg, Germany).

### Metabolite measurements

The NADP/NADPH-Glo^TM^ assay (Promega, Madison, WI) was used to detect total oxidized and reduced nicotinamide adenine dinucleotide phosphates. The ROS-Glo^TM^ H_2_O_2_ assay (Promega, Madison, WI) was used to measure the level of hydrogen peroxide (H_2_O_2_) in culture. All assays were performed as directed in the protocol. Briefly, for NADP/NADPH measurements, tumor cells were seeded at the same concentration (10^4^ per well) in triplicate in 24 well plates for 6 hours prior to the addition of an equal volume of the NADP/NADPH-Glo ^TM^ detection reagent to each well. The luminescence is read after incubation with the detection reagent for 60 min at room temperature. For H_2_O_2_ measurements, tumor cells were seeded at the same concentration (10^4^ per well) in triplicate in 24 well plates for 24 hours (aerobic incubator). 25μM of the H_2_O_2_ substrate was added for the last 6 hours of incubation and 50μl of media samples per well were removed and mixed with detection solution (50μl). Luminescence was measured after incubation with the detection solution for 20 minutes at room temperature.

## SUPPLEMENTARY MATERIAL FIGURES AND TABLES


