# Supplementary material for: Proteomic profiling of high risk medulloblastoma reveals functional biology
Source: Oncotarget. 2015 Apr 23;6(16):14584–95. doi: 10.18632/oncotarget.3927 (PMC4546489; doi:10.18632/oncotarget.3927)
Supplement: Supplementary file 1 [file oncotarget-06-14584-s001.pdf]

Proteomic profiling of high risk medulloblastoma reveals functional biology

Supplementary Material

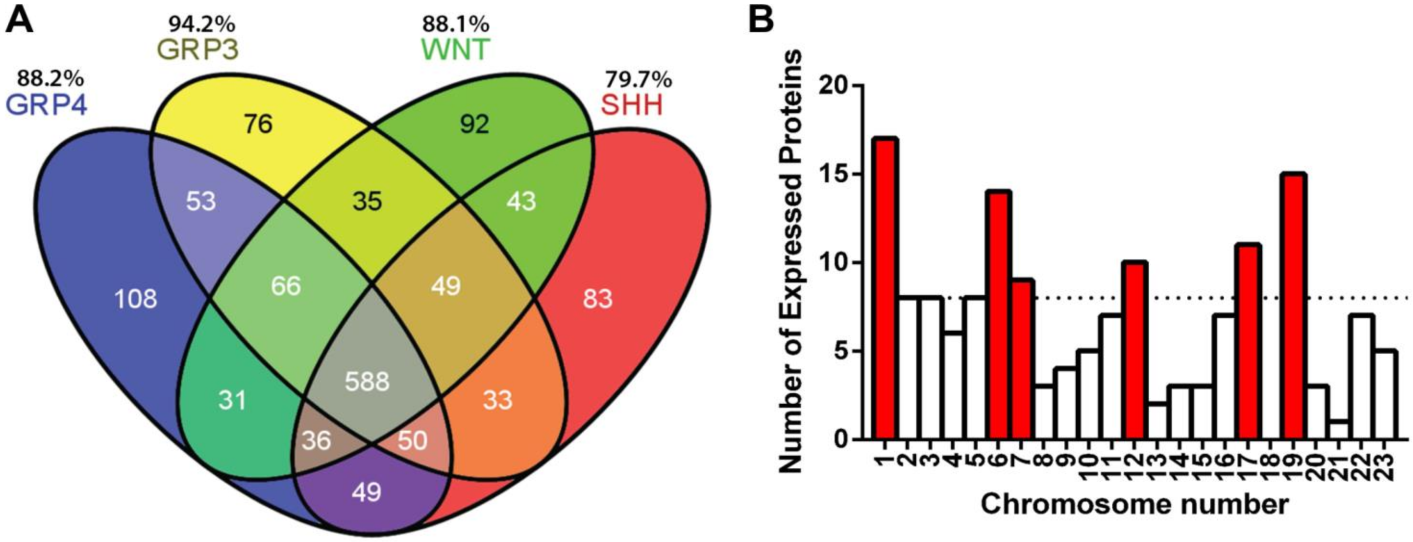

FigureS1: Protein expression profile across all MB subgroups. **A**; Venn diagram showing the number of proteins shared across the MB subgroups (the percentages above each subgroup indicate our super-SILAC reference coverage i.e. the number of sample peptides with matched heavy labeled references). **B**; The chromosomal location of the top 100 differentially expressed proteins across all subgroups (red columns indicate well known chromosomal aberrations associated with MB).

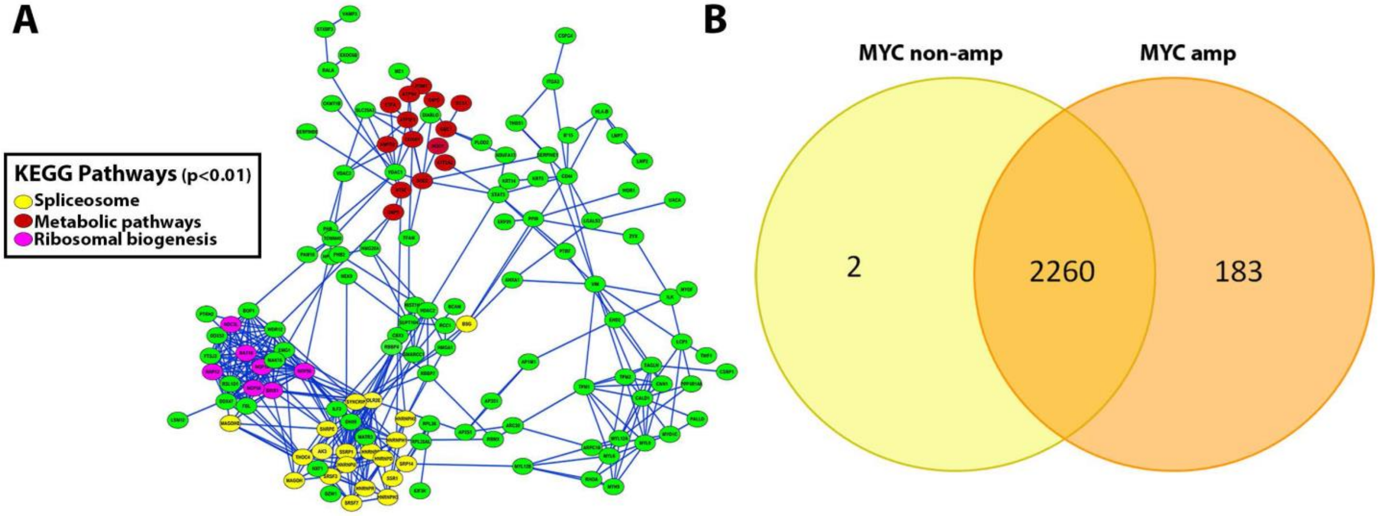

FigureS2: Differential protein expression pattern and Kegg pathway analysis between MYC-amplified and non-amplified tumors. **A**; Most significant predicted Kegg pathways when using only differentially expressed proteins (>2 fold and p<0.05) between MYC-amplified versus non-amplified Group 3 MBs. **B**; Venn diagram showing significant (p<0.05, all >6 fold) proteins expression between MYC-amplified and non-amplified tumors.

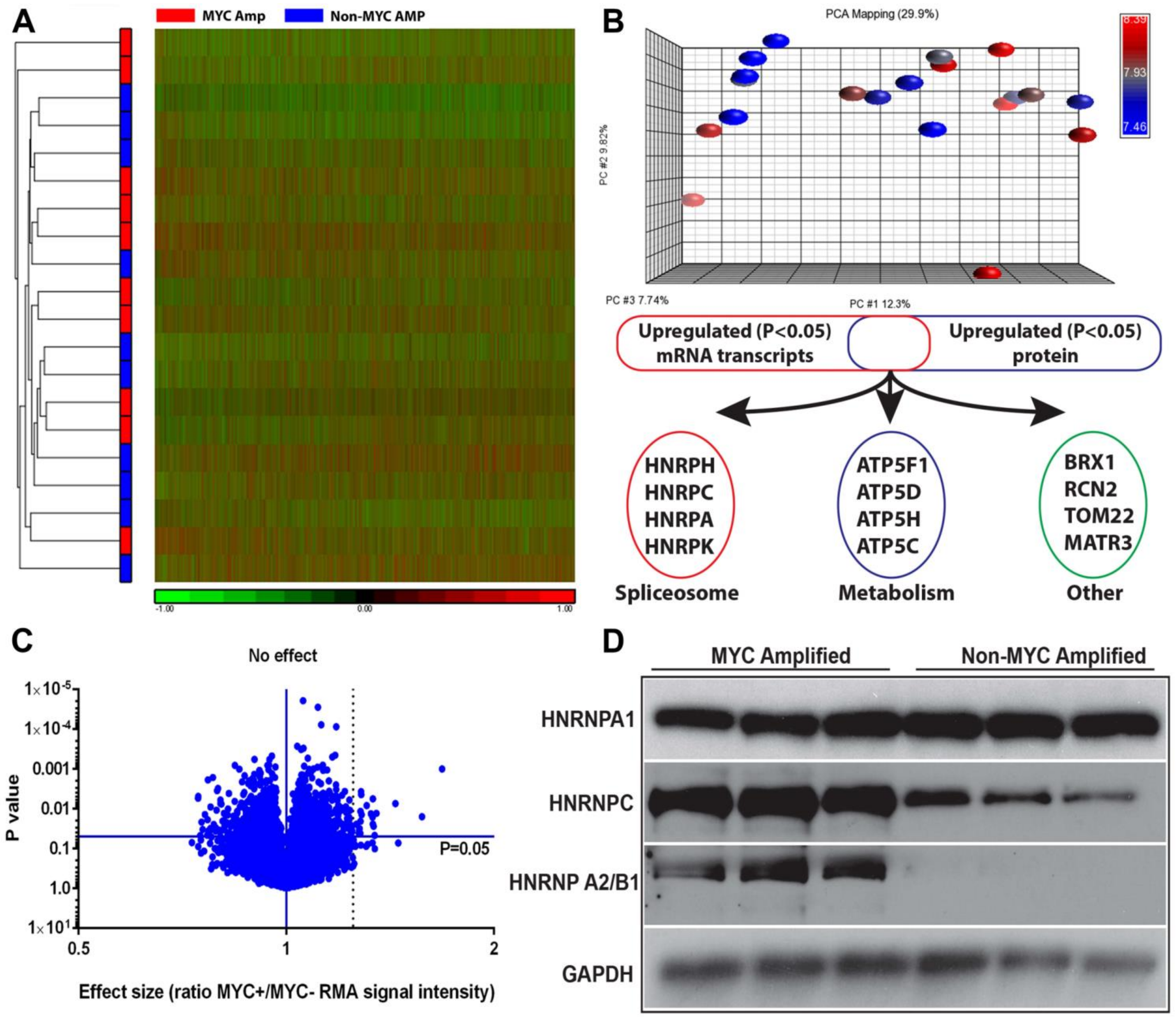

FigureS3: Unsupervised hierarchical clustering and pathway analysis using human transcriptome data. **A,B**; Unsupervised clustering of gene transcriptome data from 20 Group 3 MBs and principal component analysis was unable to segregate tumors with a MYC-amplification. **C;** Although gene transcriptome analysis revealed few significant mRNA transcript differences between MYC-amplified versus non-amplified tumors, the overlap in differentially expressed proteins and mRNA were principally in pathways associated with alternative splicing, metabolism and ribosomal biogenesis. **D**; Western blot confirmation of key proteomic differences observed using mass spectrometry.

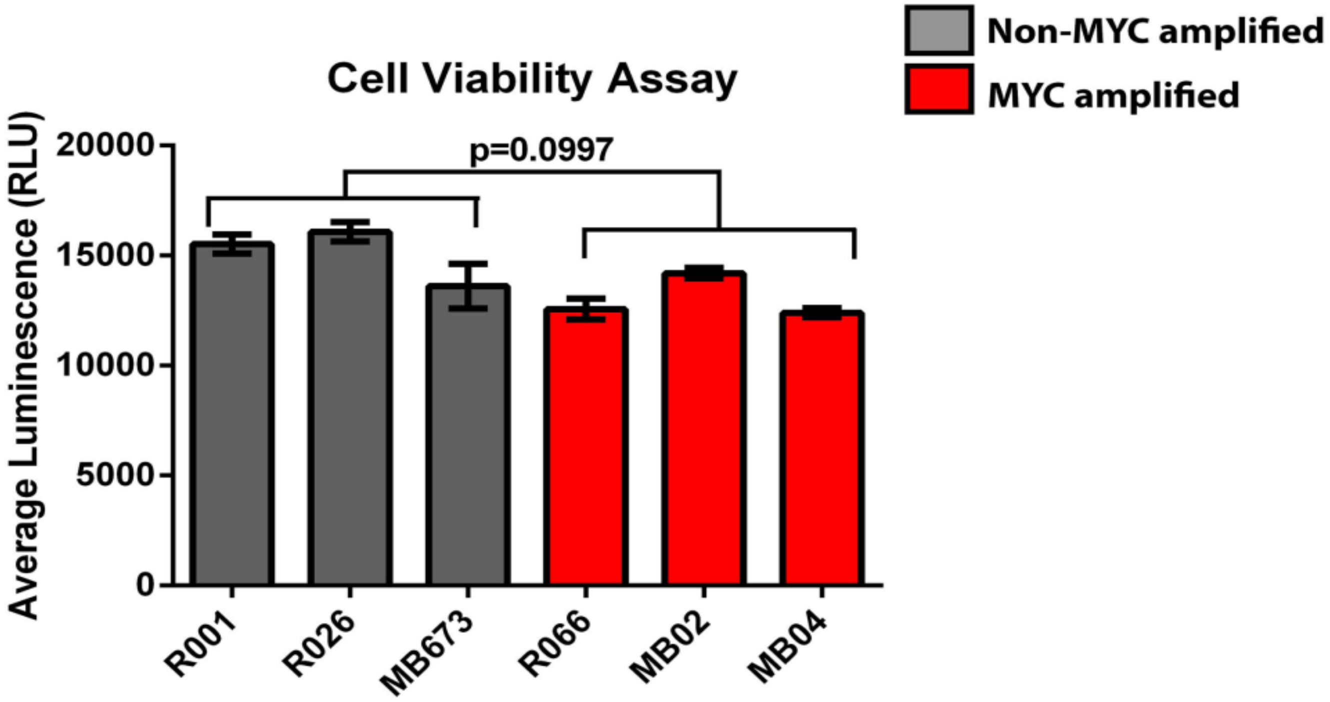

Figure S4: Cell viability measurements (using Cell Titer Glo ®) after ROS assay. No significant difference in cell density following ROS measurements.

Table S1: Differentially expressed proteins: MYC non-amplified versus MYC amplified.

LOCUS	P value	Mean1	Mean2	Difference	of differen	t ratio	df
sp|P13987|CD59_HUMAN	1.59E-07	0	0.895	-0.895	0.003727	240.154	3
sp|Q8WTT2|NOC3L_HUMAN	3.18E-06	3.30667	0.035	3.27167	0.036956	88.5289	3
sp|O95861|BPNT1_HUMAN	4.93E-06	0	0.285	-0.285	0.003727	76.4736	3
sp|P61586|RHOA_HUMAN	6.88E-06	0	0.255	-0.255	0.003727	68.4237	3
sp|P16070|CD44_HUMAN	7.3E-06	0	0.5	-0.5	0.007454	67.0821	3
sp|Q13045|FLII_HUMAN	8.79E-06	0	0.235	-0.235	0.003727	63.0572	3
sp|P35237|SPB6_HUMAN	1E-05	0	0.45	-0.45	0.007454	60.3738	3
sp|P51911|CNN1_HUMAN	1.06E-05	0	2.21	-2.21	0.037268	59.3006	3
sp|Q7L1Q6|BZW1_HUMAN	2.54E-05	0	0.165	-0.165	0.003727	44.2741	3
sp|P08729|K2C7_HUMAN	3.74E-05	0	0.29	-0.29	0.007454	38.9076	3
sp|Q96QR8|PURB_HUMAN	3.74E-05	0	0.145	-0.145	0.003727	38.9076	3
sp|O00186|STXB3_HUMAN	3.74E-05	0	0.29	-0.29	0.007454	38.9076	3
sp|Q9BUL8|PDC10_HUMAN	3.74E-05	0	0.145	-0.145	0.003727	38.9076	3
sp|P30464|1B15_HUMAN	7.01E-05	0	0.47	-0.47	0.014907	31.5285	3
sp|P30481|1B44_HUMAN	7.01E-05	0	0.47	-0.47	0.014907	31.5285	3
sp|Q99598|TSNAX_HUMAN	7.48E-05	0	0.115	-0.115	0.003727	30.8577	3
sp|Q01995|TAGL_HUMAN	9.6E-05	0.043333	0.905	-0.861667	0.030353	28.3883	3
sp|Q96EK6|GNA1_HUMAN	9.82E-05	0	0.105	-0.105	0.003727	28.1745	3
sp|Q92572|AP3S1_HUMAN	9.82E-05	0	0.21	-0.21	0.007454	28.1745	3
sp|Q4VC31|CCD58_HUMAN	9.82E-05	0	0.105	-0.105	0.003727	28.1745	3
sp|Q8N6L1|KTAP2_HUMAN	9.82E-05	0	0.315	-0.315	0.01118	28.1745	3
sp|O95678|K2C75_HUMAN	0.000118	0.006667	5.045	-5.03833	0.190261	26.4812	3
sp|Q16576|RBBP7_HUMAN	0.000132	0	0.095	-0.095	0.003727	25.4912	3
sp|O43617|TPPC3_HUMAN	0.000132	0	0.19	-0.19	0.007454	25.4912	3
sp|Q96A00|PP14A_HUMAN	0.000174	0	3.64	-3.64	0.156525	23.2551	3
sp|P28065|PSB9_HUMAN	0.000185	0	0.17	-0.17	0.007454	22.8079	3
sp|Q5T4S7|UBR4_HUMAN	0.000185	0	0.17	-0.17	0.007454	22.8079	3
sp|P13807|GYS1_HUMAN	0.000208	0	0.245	-0.245	0.01118	21.9135	3
sp|P30043|BLVRB_HUMAN	0.000208	0	0.245	-0.245	0.01118	21.9135	3
sp|Q9NRX4|PHP14_HUMAN	0.000243	0	0.31	-0.31	0.014907	20.7954	3
sp|Q6IAA8|LTOR1_HUMAN	0.000248	0	0.385	-0.385	0.018634	20.6613	3
sp|P28062|PSB8_HUMAN	0.000268	0	0.15	-0.15	0.007454	20.1246	3
sp|O00461|GOLI4_HUMAN	0.000329	0	0.14	-0.14	0.007454	18.783	3
sp|Q6NZI2|PTRF_HUMAN	0.00034	0.033333	0.905	-0.871667	0.046894	18.5879	3
sp|Q9Y3U8|RL36_HUMAN	0.000411	0	0.065	-0.065	0.003727	17.4413	3
sp|Q969Q0|RL36L_HUMAN	0.000411	0	0.13	-0.13	0.007454	17.4413	3
sp|Q9Y5B9|SP16H_HUMAN	0.000487	1.39333	0.03	1.36333	0.082776	16.4702	3
sp|A0FGR8|ESYT2_HUMAN	0.000507	0	0.545	-0.545	0.033541	16.2488	3
sp|Q04446|GLGB_HUMAN	0.000514	0.01	0.705	-0.695	0.042979	16.1706	3
sp|O75436|VP26A_HUMAN	0.000567	0	0.175	-0.175	0.01118	15.6525	3
sp|Q9BXS5|AP1M1_HUMAN	0.000567	0	0.175	-0.175	0.01118	15.6525	3
sp|Q9Y2D4|EXC6B_HUMAN	0.000567	0	0.35	-0.35	0.022361	15.6525	3
sp|P40616|ARL1_HUMAN	0.000675	0	0.22	-0.22	0.014907	14.7581	3
sp|P10301|RRAS_HUMAN	0.000753	0	0.795	-0.795	0.055902	14.2214	3
sp|Q9BXY0|MAK16_HUMAN	0.000783	2.75333	0	2.75333	0.19612	14.039	3
sp|Q15836|VAMP3_HUMAN	0.000858	0	0.355	-0.355	0.026088	13.6081	3

sp|P11172|UMPS_HUMAN	0.000895	0	0.1	-0.1	0.007454	13.4164	3
sp|P40763|STAT3_HUMAN	0.000895	0	0.2	-0.2	0.014907	13.4164	3
sp|Q01433|AMPD2_HUMAN	0.00099	0	0.145	-0.145	0.01118	12.9692	3
sp|O75083|WDR1_HUMAN	0.001042	0	0.38	-0.38	0.029814	12.7456	3
sp|Q9UI15|TAGL3_HUMAN	0.001222	0.02	0.335	-0.315	0.026088	12.0748	3
sp|Q13418|ILK_HUMAN	0.001292	0	0.53	-0.53	0.044721	11.8512	3
sp|P21291|CSRP1_HUMAN	0.001682	0.043333	0.545	-0.501667	0.046298	10.8356	3
sp|Q9BZF9|UACA_HUMAN	0.001729	0.06	1.16	-1.1	0.102469	10.7349	3
sp|Q08945|SSRP1_HUMAN	0.00177	1.56	0.05	1.51	0.141814	10.6478	3
sp|P46939|UTRO_HUMAN	0.001772	0	0.595	-0.595	0.055902	10.6437	3
sp|Q9NZM1|MYOF_HUMAN	0.001794	0.043333	0.91	-0.866667	0.081763	10.5997	3
sp|Q05682|CALD1_HUMAN	0.001875	0.15	1.1	-0.95	0.090982	10.4416	3
sp|P46379|BAG6_HUMAN	0.00196	0	0.115	-0.115	0.01118	10.2859	3
sp|P35908|K22E_HUMAN	0.002042	0	7.03	-7.03	0.693181	10.1416	3
sp|Q8WWI1|LMO7_HUMAN	0.002144	0	0.855	-0.855	0.085716	9.97481	3
sp|P36871|PGM1_HUMAN	0.00217	0.033333	0.475	-0.441667	0.044462	9.93362	3
sp|Q9BQE5|APOL2_HUMAN	0.002308	0	0.29	-0.29	0.029814	9.72689	3
sp|P05121|PAI1_HUMAN	0.002691	0.05	0.96	-0.91	0.098601	9.22909	3
sp|Q96A72|MGN2_HUMAN	0.002809	2.59667	0.08	2.51667	0.27672	9.09463	3
sp|P61326|MGN_HUMAN	0.002865	2.6	0.085	2.515	0.278413	9.03334	3
sp|P80723|BASP1_HUMAN	0.003095	4.62	0.315	4.305	0.489401	8.79646	3
sp|Q3MHD2|LSM12_HUMAN	0.003174	0	0.13	-0.13	0.014907	8.72066	3
sp|P35579|MYH9_HUMAN	0.003362	0.2	0.77	-0.57	0.066667	8.55	3
sp|P13073|COX41_HUMAN	0.003502	2.82333	0.145	2.67833	0.317681	8.43089	3
sp|Q9BRF8|CPPED_HUMAN	0.003589	0	0.405	-0.405	0.048448	8.35945	3
sp|P32243|OTX2_HUMAN	0.00385	9.00667	0.07	8.93667	1.09528	8.15928	3
sp|P48163|MAOX_HUMAN	0.004416	0	0.145	-0.145	0.018634	7.78152	3
sp|P45880|VDAC2_HUMAN	0.004488	2.51333	0.175	2.33833	0.302176	7.73832	3
sp|P07910|HNRPC_HUMAN	0.004644	2.83667	0.085	2.75167	0.359829	7.64715	3
sp|P17096|HMGA1_HUMAN	0.005017	5.81333	0.06	5.75333	0.772813	7.44466	3
sp|O14558|HSPB6_HUMAN	0.005251	0	0.355	-0.355	0.048448	7.32742	3
sp|O00469|PLOD2_HUMAN	0.005823	0.03	0.305	-0.275	0.038909	7.06782	3
sp|P50895|BCAM_HUMAN	0.0059	0	1.075	-1.075	0.152798	7.03543	3
sp|Q9H0A0|NAT10_HUMAN	0.006407	3.89333	0.05	3.84333	0.562254	6.83558	3
sp|P36957|ODO2_HUMAN	0.006468	1.76	0.12	1.64	0.240717	6.81299	3
sp|Q96DB5|RMD1_HUMAN	0.00676	0	0.125	-0.125	0.018634	6.7082	3
sp|O15143|ARC1B_HUMAN	0.00676	0	0.275	-0.275	0.040995	6.7082	3
sp|P35613|BASI_HUMAN	0.006959	2.69333	0.165	2.52833	0.380758	6.64026	3
sp|Q15942|ZYX_HUMAN	0.007025	0.016667	0.26	-0.243333	0.036768	6.61816	3
sp|Q9NZN4|EHD2_HUMAN	0.007122	0.036667	0.725	-0.688333	0.104505	6.58661	3
sp|Q92820|GGH_HUMAN	0.007389	0	0.315	-0.315	0.048448	6.5018	3
sp|Q9Y2R4|DDX52_HUMAN	0.007654	3.56333	0	3.56333	0.554894	6.42165	3
sp|Q14137|BOP1_HUMAN	0.008085	3.3	0	3.3	0.523928	6.29858	3
sp|Q6UVK1|CSPG4_HUMAN	0.008506	0.1	0.9	-0.8	0.129314	6.18647	3
sp|Q08211|DHX9_HUMAN	0.008713	2.32	0.09	2.23	0.363547	6.134	3
sp|Q5JTH9|RRP12_HUMAN	0.008887	2.97333	0.04	2.93333	0.481568	6.09122	3
sp|Q9Y314|NOSIP_HUMAN	0.009112	0	0.09	-0.09	0.014907	6.03738	3

sp|O96008|TOM40_HUMAN	0.010498	2.32667	0.045	2.28167	0.397463	5.74058	3
sp|P04083|ANXA1_HUMAN	0.010551	0.05	0.28	-0.23	0.040139	5.73014	3
sp|P37108|SRP14_HUMAN	0.010723	1.82333	0.065	1.75833	0.308633	5.69716	3
sp|Q9UBQ5|EIF3K_HUMAN	0.010855	2.80333	0.06	2.74333	0.48364	5.67227	3
sp|Q9GZL7|WDR12_HUMAN	0.011197	3.31667	0	3.31667	0.591248	5.6096	3
sp|P18754|RCC1_HUMAN	0.011208	2.83	0.02	2.81	0.50111	5.60755	3
sp|P13804|ETFA_HUMAN	0.011937	2.47	0.22	2.25	0.41042	5.48219	3
sp|Q09028|RBBP4_HUMAN	0.012095	2.20667	0.085	2.12167	0.388843	5.45635	3
sp|P07196|NFL_HUMAN	0.012502	0.076667	0.345	-0.268333	0.049768	5.39169	3
sp|Q9UBU9|NXF1_HUMAN	0.012654	2.33667	0	2.33667	0.435273	5.36827	3
sp|Q9UHB6|LIMA1_HUMAN	0.012665	0	0.16	-0.16	0.029814	5.36656	3
sp|P43307|SSRA_HUMAN	0.012857	1.29667	0.16	1.13667	0.21296	5.33747	3
sp|P08670|VIME_HUMAN	0.013337	0.093333	0.32	-0.226667	0.043033	5.26726	3
sp|P12532|KCRU_HUMAN	0.013475	3.09	0	3.09	0.588831	5.24768	3
sp|Q12792|TWF1_HUMAN	0.015132	0	0.15	-0.15	0.029814	5.03115	3
sp|Q86TI2|DPP9_HUMAN	0.015132	0	0.15	-0.15	0.029814	5.03115	3
sp|P02533|K1C14_HUMAN	0.015549	0.576667	11.51	-10.9333	2.19476	4.98157	3
sp|Q9NR28|DBLOH_HUMAN	0.016192	2.32333	0.1	2.22333	0.452974	4.90831	3
sp|P04179|SODM_HUMAN	0.016297	1.73667	0.08	1.65667	0.338324	4.89669	3
sp|P31942|HNRH3_HUMAN	0.016427	3.93	0.075	3.855	0.789559	4.88247	3
sp|O75915|PRAF3_HUMAN	0.016457	3.34333	0.14	3.20333	0.65652	4.87926	3
sp|Q8TD19|NEK9_HUMAN	0.017291	0	0.125	-0.125	0.026088	4.79157	3
sp|Q9Y3D7|TIM16_HUMAN	0.017291	0	0.125	-0.125	0.026088	4.79157	3
sp|O76021|RL1D1_HUMAN	0.018012	3.10667	0.07	3.03667	0.643356	4.72004	3
sp|Q9H0S4|DDX47_HUMAN	0.018688	2.32667	0	2.32667	0.499685	4.65627	3
sp|O95202|LETM1_HUMAN	0.019616	2.88333	0	2.88333	0.630447	4.57348	3
sp|Q9P0J0|NDUAD_HUMAN	0.020703	2.22333	0.115	2.10833	0.470318	4.48278	3
sp|Q16629|SRSF7_HUMAN	0.020745	3.62	0.055	3.565	0.795866	4.4794	3
sp|P53680|AP2S1_HUMAN	0.020835	0	0.5	-0.5	0.111803	4.47214	3
sp|P15559|NQO1_HUMAN	0.020987	0	0.615	-0.615	0.137891	4.46005	3
sp|P07951|TPM2_HUMAN	0.021066	0.2	0.795	-0.595	0.133593	4.45381	3
sp|O00159|MYO1C_HUMAN	0.021499	0.083333	0.56	-0.476667	0.107841	4.4201	3
sp|Q8WX93|PALLD_HUMAN	0.021733	0	1.05	-1.05	0.238514	4.40226	3
sp|P26006|ITA3_HUMAN	0.021814	0.116667	0.835	-0.718333	0.163399	4.3962	3
sp|P13645|K1C10_HUMAN	0.021994	1.45667	11.51	-10.0533	2.29387	4.38269	3
sp|P38159|RBMX_HUMAN	0.022812	3.54	0.08	3.46	0.800347	4.32312	3
sp|P51648|AL3A2_HUMAN	0.023048	2.04667	0.05	1.99667	0.463641	4.30649	3
sp|Q8N5M9|JAGN1_HUMAN	0.023355	3.27667	0.045	3.23167	0.754158	4.28513	3
sp|P21589|5NTD_HUMAN	0.023656	0	0.89	-0.89	0.2087	4.2645	3
sp|P13647|K2C5_HUMAN	0.023682	0	6.99	-6.99	1.63978	4.26276	3
sp|Q00839|HNRPU_HUMAN	0.024004	2.44	0.055	2.385	0.562349	4.24114	3
sp|Q00059|TFAM_HUMAN	0.024366	4.85667	0	4.85667	1.15163	4.21723	3
sp|Q13185|CBX3_HUMAN	0.024727	3.02667	0.07	2.95667	0.704995	4.19388	3
sp|P09493|TPM1_HUMAN	0.025189	0.16	0.675	-0.515	0.123659	4.16466	3
sp|P19105|ML12A_HUMAN	0.025679	0.383333	0.675	-0.291667	0.070547	4.13437	3
sp|Q14103|HNRPD_HUMAN	0.026663	2.87333	0.09	2.78333	0.6829	4.07576	3
sp|Q8IY81|RRMJ3_HUMAN	0.026934	2.61667	0.085	2.53167	0.623546	4.06011	3

sp|P11233|RALA_HUMAN	0.027138	2.86	0.14	2.72	0.671855	4.04849	3
sp|Q96QC0|PP1RA_HUMAN	0.027557	0	0.06	-0.06	0.014907	4.02492	3
sp|Q3KQU3|MA7D1_HUMAN	0.027557	0	0.18	-0.18	0.044721	4.02492	3
sp|P16615|AT2A2_HUMAN	0.027607	3.97333	0.085	3.88833	0.966741	4.0221	3
sp|O60506|HNRPQ_HUMAN	0.029071	2.22	0.08	2.14	0.542679	3.9434	3
sp|P31943|HNRH1_HUMAN	0.029181	2.41333	0.09	2.32333	0.590025	3.93769	3
sp|P23284|PPIB_HUMAN	0.029862	1.68667	0.18	1.50667	0.386029	3.90299	3
sp|P84103|SRSF3_HUMAN	0.029935	3.03	0.05	2.98	0.764235	3.89932	3
sp|O14950|ML12B_HUMAN	0.030209	0.393333	0.675	-0.281667	0.072489	3.88566	3
sp|P24539|AT5F1_HUMAN	0.030354	3.29	0.17	3.12	0.804432	3.87851	3
sp|P49755|TMEDA_HUMAN	0.031083	2.39333	0.19	2.20333	0.573311	3.84317	3
sp|P13796|PLSL_HUMAN	0.035702	0	0.095	-0.095	0.026088	3.6416	3
sp|Q9Y2X3|NOP58_HUMAN	0.036345	4.23667	0.065	4.17167	1.15361	3.61619	3
sp|P60660|MYL6_HUMAN	0.036406	0.346667	0.675	-0.328333	0.090855	3.61382	3
sp|O75340|PDCD6_HUMAN	0.036555	2.22	0.165	2.055	0.569564	3.60803	3
sp|O00567|NOP56_HUMAN	0.036776	4.64	0	4.64	1.28908	3.59947	3
sp|Q92769|HDAC2_HUMAN	0.036779	2.13333	0.04	2.09333	0.581585	3.59936	3
sp|P22087|FBRL_HUMAN	0.036787	5.7	0.045	5.655	1.57126	3.59903	3
sp|P55795|HNRH2_HUMAN	0.037171	2.25333	0.095	2.15833	0.602153	3.58436	3
sp|O43390|HNRPR_HUMAN	0.03783	4.39667	0.065	4.33167	1.21689	3.55963	3
sp|Q9UIJ7|KAD3_HUMAN	0.038001	1.74	0.335	1.405	0.395408	3.55329	3
sp|Q86V81|THOC4_HUMAN	0.038661	3.19	0.055	3.135	0.888296	3.52923	3
sp|P07996|TSP1_HUMAN	0.039427	0.13	0.735	-0.605	0.172763	3.5019	3
sp|Q92922|SMRC1_HUMAN	0.039441	1.41667	0.175	1.24167	0.354619	3.50141	3
sp|P62304|RUXE_HUMAN	0.039883	2.47333	0	2.47333	0.709512	3.48596	3
sp|Q8TDN6|BRX1_HUMAN	0.04013	2.88667	0.245	2.64167	0.759663	3.47742	3
sp|O75947|ATP5H_HUMAN	0.0402	3.68667	0.18	3.50667	1.00911	3.47502	3
sp|P35232|PHB_HUMAN	0.040491	2.53667	0.09	2.44667	0.706098	3.46505	3
sp|P24844|MYL9_HUMAN	0.04055	0.133333	0.73	-0.596667	0.172294	3.46307	3
sp|Q12905|ILF2_HUMAN	0.041031	2.73	0.07	2.66	0.771722	3.44683	3
sp|P43243|MATR3_HUMAN	0.041369	2.67	0.07	2.6	0.756784	3.43559	3
sp|Q9Y3C1|NOP16_HUMAN	0.041444	2.18667	0	2.18667	0.636934	3.43311	3
sp|P62805|H4_HUMAN	0.04203	4.59333	0.08	4.51333	1.32204	3.41391	3
sp|P30040|ERP29_HUMAN	0.042499	1.49667	0.18	1.31667	0.387394	3.39878	3
sp|Q92979|NEP1_HUMAN	0.043524	3.91667	0	3.91667	1.16343	3.36648	3
sp|P21796|VDAC1_HUMAN	0.043823	3.02	0.165	2.855	0.8504	3.35724	3
sp|P59998|ARPC4_HUMAN	0.044135	1.64	0.3	1.34	0.400278	3.34768	3
sp|P17931|LEG3_HUMAN	0.044888	0	0.57	-0.57	0.171432	3.32494	3
sp|Q99623|PHB2_HUMAN	0.04516	2.61667	0.085	2.53167	0.763275	3.31685	3
sp|P37235|HPCL1_HUMAN	0.045315	3.44	0.26	3.18	0.960064	3.31228	3
sp|Q00325|MPCP_HUMAN	0.046129	2.29	0.21	2.08	0.632499	3.28854	3
sp|P11413|G6PD_HUMAN	0.046568	0.036667	0.25	-0.213333	0.065121	3.27595	3
sp|Q9NP66|HM20A_HUMAN	0.048257	3.08667	0	3.08667	0.955956	3.22888	3
sp|P19388|RPAB1_HUMAN	0.048893	2.47	0.12	2.35	0.731703	3.21169	3
sp|Q9Y3E5|PTH2_HUMAN	0.049867	3.55667	0.13	3.42667	1.07557	3.18591	3

Table S2: Human tissue sample details.

SAMPLE ID	GENDER	AGE AT SURGERY (YEARS)	HISTOLOGY	METASTATIC STATUS	450K ARRAY	NANOSTRING	MYC STATUS	SOURCE LAB
A00363	F	10.5	Normal cerebellum					CNMC
A197	M	1	Normal cerebellum					CNMC
A198	F	6	Cortical dysplasia					CNMC
NCPM1	M	0	Normal cerebellum					CNMC
698	M	2.3	Classic	M0 (Became M1 3 months into therapy)		Group3	Non-amp FISH	CNMC
673	M	7.2y	Classic	M0		Group3	Non-amp FISH	CNMC
Med57	M	2.8y	Classic	M3	Group3		Non-amp PCR	CNMC
Med69	M	7.5y	Classic	M0	Group3	Group3	Non-amp PCR	CNMC
A610	M	12y	Large Cell Anaplastic	M0	Group3	Group3	Non-amp FISH	CNMC
A541	M	4.1y	Classic	M1	Group3	Group3	Non-amp FISH	CNMC
Med63	M	4.8y	Classic	M0	Group4	Group3	Non-amp PCR	CNMC
Med53	M	0.8yrs	Medullo NOS	M3	Group3/4		Non-amp PCR	CNMC
MDT-MB-1166	F	7	Large Cell Anaplastic	M0		Group3	Non-amp PCR	Toronto
MDT-MB-1219	unknown	7	Melanotic differentiation	unknown		Group3	MYC-amp PCR and 450K	Toronto
R001	M	5.2	Classic	M+		Group3	Non-amp FISH	QCMRI
R066	M	5.5	Classic with anaplasia	M+		Group3	MYC-amp FISH	QCMRI
R026	F	6.9	Classic	M0		Group3	Non-amp FISH	QCMRI
MDT-MB-1364	M	11	Large Cell Anaplastic	M0		Group3	gain of chromosome arm	Toronto
MDT-MB-1402	M	1	Large Cell Anaplastic	unknown		Group3	Non-amp PCR	Toronto
MB90	M	1.6	Large Cell Anaplastic	M0	Group3		Non-amp PCR	DKFZ
MB95	M	3	Classic	M0	Group3		MYC-amp PCR	DKFZ

**MB18**	**F**	**13**	**Large Cell Anaplastic**	**M0**	**Group3**		**Non-amp PCR**	**DKFZ**

CNMC – Children's National Medical Center
DKFZ – German Cancer Research Center
QCMRI – Queensland Children's Medical Research Institute
